# Effect of religiosity/spirituality and sense of coherence on depression within a rural population in Greece: the Spili III project

**DOI:** 10.1186/s12888-015-0561-3

**Published:** 2015-07-25

**Authors:** Dimitrios Anyfantakis, Emmanouil K. Symvoulakis, Manolis Linardakis, Sue Shea, Demosthenes Panagiotakos, Christos Lionis

**Affiliations:** Clinic of Social and Family Medicine, School of Medicine, University of Crete, Heraklion, Crete Greece; Preventive Medicine & Nutrition Clinic, School of Medicine, University of Crete, P.O. Box 2208, Heraklion, Crete Greece; Department of Nutrition-Dietetics, Harokopio University, Athens, Greece

**Keywords:** Beliefs, Religious, Spirituality, Sense of coherence, Depression

## Abstract

**Background:**

Recent research has addressed the hypothesis that religiosity/spirituality and sense of coherence buffer the negative effects of stress on numerous health issues. The aim of the current study was to further this work by exploring potential links between psycho-social factors such as religiosity/spirituality and sense of coherence with depression.

**Methods:**

A total number of 220 subjects of the SPILI III cohort (1988–2012) attending a primary care setting in the town of Spili on rural Crete represented the target group. All participants underwent a standardized procedure. Validated questionnaires were used to evaluate sense of coherence, depression levels and religious and spiritual beliefs. A multiple linear regression analysis of the Beck Depression Inventory Scale (BDI) in relation to demographic characteristics, scores on the Royal Free Interview for Spiritual and Religious Beliefs scale (RFI-SRB) and Sense of Coherence scale (SOC) was performed.

**Results:**

A significant inverse association was found between BDI and RFI-SRB scale (B-coef = −0.6999, *p* < 0.001), as well as among BDI and SOC scale (B-coef = −0.556, *p* < 0.001).

**Conclusions:**

The findings of the current observational study indicate that highly religious participants are less likely to score high in the depression scale. Furthermore, participants with high SOC scored significantly lower in the BDI scale. Further research is required in order to explore the potential effect of SOC and religiosity/spirituality on mental health.

**Electronic supplementary material:**

The online version of this article (doi:10.1186/s12888-015-0561-3) contains supplementary material, which is available to authorized users.

## Background

Convincing scientific evidence outlines the important role of psychosocial factors on the pathogenesis of coronary artery disease [1]. Remarkably, chronic stress conditions, depression, anxiety and social isolation have been reported to exert direct atherogenic effects through hypercortisolemia and sympathetic overdrive. Consequently this can lead to a variety of peripheral manifestations ranging from blood pressure and heart rate elevation to direct damage on vascular endothelium [1]. On account of these findings, Rozanski et al., describe a model based on the study of positive psychosocial factors that may amplify the above previously reported adverse neuro-hormonal reactions [2]. Likewise,, data from a recent literature review link positive psychological factors such as optimism and hedonic well being with a reduced risk of cardiovascular events [3]. Metabolic and inflammatory processes have also been considered among others responsible for these associations [3]. Furthermore, optimism, defined as the generalized expectation that good things will occur in one’s life, is inversely associated with anxiety and depression, and is generally considered a positive health determinant during periods of increased stress [4].

One theoretical framework which can offer some plausible mechanisms to explain certain research findings, is that of sense of coherence (SOC). SOC, the principal concept of salutogenesis introduced by Antonovsky in 1987 [5], consists of an important health promoting resource that induces a positive perceived state of well being [6]. The salutogenic orientation attempts to explain why some people who encounter major stress remain well while others do not [5]. According to this model, it would appear that individuals with a strong SOC cope better with stress, which in turn has a favorable effect on health [6]. More specifically, SOC could be defined as the global orientation, which reflects the extent to which one has a pervasive, enduring though dynamic feeling of confidence that the stimuli deriving from one’s internal or external environments throughout life are structured, predictable and explicable; resources are available for individuals tomeet the demands posed by the stimuli; and (3) these demands are challenges, worthy of investment and engagement. According to Antonovsky, the SOC scale is composed of three sub-components: comprehensibility (the cognitive component); manageability (the behavioural component) and meaningfulness (the motivational component) [5].

Within this framework, a recent observational study in Greece was designed to examine the potential association between SOC and diabetes mellitus [7]. This study observed hospitalized patients and interestingly the findings showed that participants without diabetes were more likely to present a high SOC score compared to participants with diabetes [7].

In alignment with SOC, a growing body of research outlines the importance of religious and spiritual beliefs and practices as a coping mechanism in stressful situations [8]. It has been suggested that involvement in worship activities creates positive emotions and the sense of group belonging, which may lead to positive health outcomes [9]. Furthermore, in recent years emphasis has been attributed to the assessment of patients’ spirituality as part of the bio-psychosocial model of primary care. [10]. Recent research involving a rural population in the village of Spili on the island of Crete, Greece, examined the impact of religious and spiritual beliefs on a variety of clinical and biological markers [11]. Spili is located at an altitude of 430 m and 28 km south-east from the town of Rethymnon. The local economy of this area is based on agriculture and animal husbandry. The social network of this society is characterized by tradition and family values. The existence of numerous churches reflects the religiousness of the population.

This study, named the SPILI III study, aimed to explore to what extent psychosocial factors, such as religiosity/spirituality (R/S) and SOC, mitigate the negative effects of stress on a variety of biochemical, clinical and imaging indicators [11]. Participants with higher levels of religious and spiritual beliefs presented lower prevalence of diabetes and lower levels of intimae media thickness, while a positive association was found between SOC and R/S [11]. On the basis of these findings, the authors suggest a positive influence of R/S on several cardio-metabolic determinants underlining the necessity of further research in this area [11].

The concomitant examination of positive psycho-social determinants in conjunction with health-related issues has also been reported in the literature but it has not received sufficient attention in Greece until recently [12], especially during the austerity period. Evidence from a meta-analysis of 147 studies suggests a negative association between religiosity and depression [13]. Similar results were indicated in a cross sectional cohort study among patients with advanced illness, which aimed to examine the association of spiritual well-being with symptoms of anxiety and depression [14]. One of the main findings of this research was that following adjustments, greater spiritual well-being was associated with fewer depressive symptoms (*p* < 0.001) [14].

Thus, certain questions that have been addressed in other national settings still remain unanswered in Greece. This subject attracts additional interest at a time when there is much discussion regarding the co-morbidity of depression and mental health disorders with various physical diseases, including diabetes [15] and in a period where Greek People are struggling from the past years of economic crisis. [11]. As such, the current study aimed to investigate the impact of R/S and SOC on the Beck Depression Inventory (BDI) scale with the assumption that highly religious participants would present a more favorable mental health profile . Therefore, we proceeded to examine to what extent high levels of R/S were associated with scores on the BDI scale, amongst the participating individuals.

## Methods

### Description of setting

The Spili III study represents the second follow up of a cohort study, originally established in 1988 (SPILI I) [16]. The project was launched in September 2009 at a primary health care center located in the Cretan village of Spili and was completed over an 18-month period [11, 12].

### Ethical approval

The study was approved by the Institutional Ethics Committee of the University Hospital of Crete (No Protocol:9989/02.09.2008).

### Study design and participants

A total of 220 subjects of the SPILI III cohort represented the target group [11]. All subjects received an invitation letter and were approached by telephone [11]. A total number of 195 participants (97 males, 98 females), mean age 67.2 ± 15.2 (no significant gender difference, *p* = 0.054), were re-examined, representing 88.6 % of the target group [11]. Twenty-five individuals, reporting “good health”, declined our invitation [11]. The mean age of the non-participants was more likely to be lower than the mean age of the participants [11]. Clinical characteristics of the sample have been reported in a previously published paper [11].

### Instruments used to measure outcomes

Evaluation of R/S was performed using the Royal Free Interview for Spiritual and Religious Beliefs (RFI-SRB) that has been translated and validated into Greek [17, 18]. This scale focuses on the strength of faith and it is short and simple to complete [18]. Six questions with continuous responses, each scored on a 0–10 scale (total range 0–60), were used in order to calculate the strength of religious/spiritual belief [18]. Higher values of the RFI-SRB indicate increased strength of religious and spiritual faith is high [18].

Furthermore, all participants completed the Antonovsky 29-item Sense of Coherence Scale (SOC-29) [19]. The scale has been translated into Greek and validated [20]. The instrument consists of 29 items rated on a 7-point scale format with two fixed responses, ‘never’ and ‘very often’, giving a maximum score of 203 [19]. Higher scores indicate a greater SOC [19]. The questions measure all three components of the SOC scale, with 10 questions relating to manageability, 11 questions relating to comprehensibility and 8 questions relating to meaningfulness [19]. Cronbach’s alpha coefficient of the SOC scale was equally very high (a = 0.888) [11]. Partial correlation coefficient between SOC and RFI was *r* = 0.0773, *p* < 0.001 controlling for gender, age and family status [11].

Depressive symptoms were evaluated with the use of Beck’s Depression Inventory (BDI). The instrument consists of 21-items rated on a 3-point scale [21, 22]. The total score ranges from 0–63, where high scores indicate more severe depression [21, 22]. The BDI items assess the various symptoms of depression including melancholy, anhedonia, somatic symptoms, feelings of punishment, suicide thoughts [21, 22]. In addition, BDI represents one of the most frequently used self-rating questionnaires for measuring depression worldwide, with high internal consistency and high content validity [23]. Furthermore it is considered a relevant psychometric instrument for differentiating between depressed and non-depressed subjects [23, 24].

### Statistical analysis

Data were analyzed using the Statistical Package for the Social Sciences software (IBM SPSS Statistics for Windows, Version 20.0. Armonk, NY: IBM Corp). Distributions (mean values, percentiles etc.) of descriptive characteristics of participants were estimated, using analysis of variance and chi-square test detecting gender differences. Normality of different scales was detected and log_10_ transformed values were applied where iappropriate. Multiple linear regression analysis was used on BDI scale in relation to demographic characteristics, multimorbidity status, RFI-SRB and SOC scales. In the first model only demographic characteristics were used, but in the second and third models RFI-SRB and SOC scales were used separately due to their strong association. The third model was also repeated by using the three constructs of SOC (Comprehensibility, Manageability, Meaningfulness) instead of the SOC scale. As covariates gender, age, family and religion status were used. Heterogeneity was tested by Levene’s test.

Applying analysis of covariance, levels of BDI scale were compared according to medians (per gender-age specific) of RFI-SRB and SOC scales. As covariates gender, age, family status, religion and multimorbidity were used. BDI scale was used as log_10_ transformed values.

## Results

The working sample was 195 subjects (50 % female) with a mean age of 67.2 ± 15.2 years. Descriptive characteristics of the participants are presented in Table 1. More than 7 out of 10 of the participants were married (males: 91.8 %, females: 56.1 %, *p* < 0.001) while the majority (98.5 %) were Christians Orthodox. Significantly different values were found between males and females in the SOC scale (145.7 versus 136.5, respectively, *p* = 0.022) in the comprehensibility component (52.9 versus 48.0, respectively, *p* = 0.002), and in the BDI scale (8.8 versus 10.4, respectively, *p* = 0.005). With regard to multimorbidity status, more than 2 out of 10 of the participants presented two or more co-existing medical conditions (males: 25.8 %, females: 20.4 %, *p* = 0.002). Notably, a higher frequency of hypertension was registered in females (79.6 % versus 60.8 %, respectively, *p* = 0.005).

**Table 1:** Descriptive characteristic of the 195 participants of the study

			Gender		
		Total, *n* = 195	Male, *n* = 97	Female, *n* = 98	*p*-value^a^
Age, years		67.2 (15.2)^b^	65.1 (16.2)	69.2 (13.9)	0.054
Age group	≥65	61.5	58.8	64.3	0.464
Family Status	married	73.8	91.8	56.1	<0.001
	not married, divorced, widowed	26.2	8.2	43.9	
Religion	Christian Orthodox	98.5	96.9	100.0	0.121
RFI-SRB scale (range: 3–60)		35.2 (19.6)	36.1 (20.1)	34.4 (19.1)	0.798
SOC scale (range: 82–194)		141.1 (25.2)	145.7 (26.6)	136.5 (23.1)	0.022
Comprehensible		50.4 (10.9)	52.9 (11.2)	48.0 (10.2)	0.002
Manageable		48.7 (10.4)	47.5 (9.9)	49.9 (10.7)	0.103
Meaningful		42.0 (8.2)	42.9 (8.6)	41.0 (7.6)	0.107
BDI scale (range: 1–29)		9.6 (5.1)	8.8 (5.1)	10.4 (5.1)	0.005
Minimal depression symptoms		59.0	66.0	52.0	
Mild		36.9	30.9	42.9	0.138
Moderate		4.1	3.1	5.1	
Morbidity, conditions					
Hypertension		70.3	60.8	79.6	0.005
Diabetes mellitus		18.5	20.6	16.3	0.440
Cerebrovascular event		1.5	2.1	1.0	0.621
Congestive heart failure		4.1	2.1	6.1	0.279
Myocardial Infarction		3.1	5.2	1.0	0.118
Multimorbidity					
none condition		29.7	39.2	20.4	0.002
one		47.2	35.1	59.2	
two or more		23.1	25.8	20.4	

*RFI-SRB* the Royal Free Interview for Spiritual and Religious Beliefs scale, *SOC* the Sense of Coherence scale, *BDI* Beck Depression Inventory scale

^a^Analysis of variance and chi-square tests (Fisher exact test) were done between genders. Log_10_ transformed values were used for RFI-SRB, SOC and BDI scales

^b^Values are means (standard deviations) or %

Multiple linear regression analysis of BDI scale in relation to demographic characteristics and RFI-SRB or SOC is tabulated in Table 2. In addition and according to the second and third models, BDI scale presented a significant strong negative correlation with RFI-SRB (*b* = −0.699, *p* < 0.001) as well as with SOC scale (*b* = −0.556, *p* < 0.001). When we repeated regression analysis by using the three constructs of SOC, the analysis showed a strong negative correlation between BDI scale with comprehensibility (*b* = −0.278, *p* < 0.001) and manageability (*b* = −0.258, *p* < 0.001) (results not shown in table).

**Table 2:** Hierarchical multiple linear regression analysis of Beck Depression Inventory Scale (BDI) in relation to demographic characteristics and Royal Free Interview for Spiritual and Religious Beliefs scale (RFI-SRB) or Sense of Coherence scale (SOC)

	1st model			2nd model			3rd model		
Predictors	Standardized betas	t	*p*-value	Standardized betas	t	*p*-value	Standardized betas	t	*p*-value
Gender	0.153	2.51	0.013	0.178	4.17	<0.001	0.079	1.59	0.115
Age	−0.102	−1.53	0.128	0.184	3.63	<0.001	0.083	1.46	0.146
Family status	0.028	0.43	0.670	−0.007	−0.15	0.881	−0.016	−0.30	0.763
Religion	0.068	1.20	0.234	0.014	0.35	0.729	0.015	0.32	0.751
Multimorbidity	0.646	10.40	<0.001	0.175	3.22	0.002	0.307	5.13	<0.001
RFI-SRB scale				−0.699	−14.23	<0.001	-	-	-
SOC scale				-	-	-	−0.556	−10.20	<0.001
R^2^	0.41			0.72			0.62		
R^2^ adjusted	0.40			0.71			0.61		

Categorical predictors were defined as gender (1:male, 2:female), family status (1:married, 2: not married, divorced, widowed), religion (1: Christian Orthodox, 2: Christian Evangelist) and multimorbidity (1:none, 2:one, 3:two or more). BDI and RFI-SRB scale were used as log_10_ transformed values

In addition, a strong positive relationship between RFI-SRB and SOC scales was found according to univariate regression analysis (r-Spearman = 0.745) as well as negative associations of BDI with RFI-SRB (*r* = −0.766, *p* < 0.001) or SOC (*r* = −0.719, *p* < 0.001) (results not shown in table or figures).

Table 3 demonstrates that individuals with a higher level of RFI-SRB had a lower mean score on BDI (6.1 vs 11.1, *p* < 0.001) and for the SOC scale (6.5 vs 10.5, *p* < 0.001).

**Table 3:** Levels of depression scale (BDI) according to RFI-SRB and SOC scales categorization

			Depression scale - BDI		
Scales		n	Mean values	Standard Errors	*P*-value
RFI-SRB	<median^a^	97	11.1	1.0	<0.001
	≥median	98	6.1	1.0	
SOC	<median	97	10.5	1.1	<0.001
	≥median	98	6.5	1.1	

Analysis of covariance. As covariates were used gender, age, family status, religion and multimorbidity. BDI scale was used as log_10_ and means ± stand. errors are represented as antilog_10_ values

^a^Specific per gender-age medians

## Discussion

### Main findings

Τhe main finding of the current analysis is that highly religious participants in rural Crete as identified by the RFI-SRB scale, presented a lower likelihood of depression presence, as indicated by the BDI scale. We cannot claim that this is a novel finding since it is also reported by other studies. However, it adds to the existing evidence and contributes to discussions regarding the existence of specific health assets in this population [11, 12].

This subject has been extensively discussed in the literature and in a recent literature review that examined the relationship between R/S and depressive symptoms during the last 50 years. It is notable that over half of the articles included reported less depression in individuals with higher R/S [10]. In parallel with this, a cross sectional study among patients with heart failure, found a strong association between greater spiritual well-being and less depression [25].

Although the association between religiosity and health has been discussed in the framework of the SPILI project [26, 27] the data association between R/S and mental health has not received much attention until recently. Ιn Cyprus , a study among nursing students reported a negative correlation between strong R/S beliefs (measured by RFI-SRB) and depression as assessed by the BDI questionnaire [28].

Our study further contributes to the discussion of data gathered from the implementation of SOC within a rural population and it suggests that higher SOC scores are related to lower BDI scores, indicating lower frequency of depression among individuals with high SOC. Similarly, data from a Swedish cohort study among adolescent females reported that SOC scores showed significantly inverse correlations with BDI [29]. These authors suggested that the SOC scale may be an inverse measure of persistent and generalized depressive symptoms [29]. In alignment with this, recent findings from a study among patients with pure chronic obstructive pulmonary disease indicated a negative association between depression and SOC [30]. Remarkably, raising scores in SOC by one point reduced depression by 0.21 points [30]. A potential explanatory mechanism for previously reported associations could be due to the ability of all subjects to maintain their homeostasis when this is challenged from internal or external adverse stressors [31]. Interestingly the response to stress of the human organism in order to maintain its homeostasis includes both neuro-endocrine and behavioral processes [31]. We can also assume that participation in religious activities may enhance social contact, optimism and sense of group belonging resulting directly in higher SOC and less depression. This assumption has been enhanced by a recent publication that reported data from rural Crete [32]. However the extent to which a high R/S may lead to a high SOC and consequently to low depressive symptoms is still unknown based on the results of this study, but it is an interesting subject worthy of further exploration.

We may assume that religious involvement is an interesting mediator that might account for the positive effect of R/S on health status, by encouraging healthy life-style habits (avoidance of smoking and alcohol, dietary and sexual restrictions) which in the long term may decrease morbidity [8]. Among explanatory mechanisms of these results, it has been reported that religious beliefs may help patients to cope better with stressful situations by providing meaning and hope [8]. Furthermore, religious and spiritual involvement may result in healthier life style practices such as marital fidelity, and avoidance of alcohol, drugs and smoking [8].

### Strengths and limitations

The current quantitative study aims to offer support on qualitative observations, previously reported within primary care settings [10]. The SPILI III project is a general practice based study characterized by a high level of sample ‘compactness’ in terms of religious doctrine and ethnicity, a feature which may empower our initial hypothesis. We included comparisons between scale scores and not diagnostic end-points in order to avoid human or definition errors. We checked our observational data by performing both a multiply linear regression analysis and an analysis of variance. Furthermore, since longitudinal data are required to test the buffering effect on stress and to avoid measurement or definition bias, stress measurement was not among the objectives of this study. The lack of such data did not allow us to suggest that SOC might mediate the relationship between RFI-SRB scores and depression. Additionally, although we analyzed 195 subjects our sample still remains restricted. Given the fact that this study was orientated towards sociologic aspects of well-being, the effect of a genetic background currently appears unlikely. Furthermore, the cross-sectional character of the study does not allow causative explanations of the examined associations [11]. Αn additional limitation is representedby the absence of multi-community sampling. Data collection occurred at the beginning of the financial crisis which shrinks the eventuality of bias due to a worsening economic lived experience. Furthermore, religious parameters that may offer additional insights to our future work such as praying time, church attendance, confession as well as dietary habits and confession were not examined. Moreover, the fact that our study sample consisted of elderly rural residents who are known to be closely attached to religious aspects may overestimate our key findings. Furthermore, several items of the STROBE checklist such as reporting the study population, participant sampling, statistical and test methods were followed (please see Additional file 1).

### Implications for research and/or practice

This is an effort of general practice driven translational research that attempts to bridge analytical data with psycho-social meanings in terms of religiosity, spirituality, and components of individual’s sense of coherence and well-being. Physicians dealing with mental health care should take into consideration their patients’ psychosocial backgrounds by learning to measure the effect of these on both health and morbidity. This may have a particular impact in countries affected by austerity including Greece. Primary care settings should also offer this opportunity, and as this study proved, ‘indicates’ and conventional metrics can be used to extract data beyond classic explanations of what is harmful or beneficial for people’s lives.

## Conclusions

This observational study attempted to explore the link between R/S, SOC and depressive symptoms. Interestingly, R/S and SOC showed a significant inverse correlation with depressive symptoms. These findings further enhance the hypothesis available in the literature that R/S and SOC may buffer the negative effects of stress on human health while raising some additional assumptions of a plausible explanation of this association. Further research is essential in order to study the potential effect of SOC and R/S in the area of mental health.

## Additional file

Additional file 1:
**Completed STROBE checklist.**

## Abbreviations

RFI-SRB: Royal Free Interview for Spiritual and Religious Beliefs scale
BDI: Beck depression inventory
SOC: Sense of coherence
R/S: Religiosity/Spirituality
